# Diagnosing *Clostridioides difficile* infections with molecular diagnostics: multicenter evaluation of revogene *C. difficile* assay

**DOI:** 10.1007/s10096-020-03829-4

**Published:** 2020-02-15

**Authors:** Vittorio Sambri, Cécile Gateau, Silvia Zannoli, Giorgio Dirani, Jeanne Couturier, Ingrid Op den Buijs, René Roymans, Emma Hallet, Mihaela Arnold, Anja Zumoberhaus, Stanislava Steiner, Jeroen van de Bovenkamp, Martin Altwegg, Livia Berlinger, Frédéric Barbut, revogene C. Difficile study group, Johan van Broeck, Michel Delmée

**Affiliations:** 1 Operative unit of Clinical Microbiology, Regional Reference Centre for Microbiological Emergencies, S. Orsola-Malpighi University Hospital, Bologna, Italy; 2 National Reference Laboratory for C. difficile, Hôpital Saint-Antoine, Assistance Publique-Hôpitaux de Paris, INSERM U-1139, University Paris Descartes, Paris, France; 3 Laboratory of Medical Microbiology, Stichting PAMM, Veldhoven, Netherlands; 4 Great Western Hospital, Swindon, UK; 5 Present Address: Royal Devon and Exeter Hospital, Exeter, UK; 6 Lucerne Cantonal Hospital, Luzern, Switzerland; 7 Bioanalytica AG, Luzern, Switzerland; 8 Present Address: Unit of Microbiology, The Great Romagna Hub Laboratory, Pievesestina, FC Italy; 9 Present Address: DIMES, University of Bologna, Bologna, Italy

## Abstract

*Clostridioides difficile* infections are a significant threat to our healthcare system, and rapid and accurate diagnostics are crucial to implement the necessary infection prevention and control measurements. Nucleic acid amplification tests are such reliable diagnostic tools for the detection of toxigenic *Clostridioides difficile* strains directly from stool specimens. In this multicenter evaluation, we determined the performance of the revogene *C. difficile* assay. The analysis was conducted on prospective stool specimens collected from six different sites in Europe. The performance of the revogene *C. difficile* assay was compared to the different routine diagnostic methods and, for a subset of the specimens, against toxigenic culture. In total, 2621 valid stool specimens were tested, and the revogene *C. difficile* assay displayed a sensitivity/specificity of 97.1% [93.3–99.0] and 98.9% [98.5–99.3] for identification of *Clostridioides difficile* infection. Discrepancy analysis using additional methods improved this performance to 98.8% [95.8–99.9] and 99.6% [99.2–99.8], respectively. In comparison to toxigenic culture, the revogene *C. difficile* assay displayed a sensitivity/specificity of 93.0% [86.1–97.1] and 99.5% [98.7–99.9], respectively. These results indicate that the revogene *C. difficile* assay is a robust and reliable aid in the diagnosis of *Clostridioides difficile* infections.

## Introduction

*Clostridioides difficile* infections (CDI), caused by the Gram-positive, spore-forming anaerobic bacterium *Clostridioides difficile* (*C. difficile*), create a tremendous burden on our healthcare system. In Europe alone, approximately 124.000–172.000 CDI cases are identified on a yearly basis, and CDI Management attributed costs approach €3.000 million [1–4]. Furthermore, CDI remains underdiagnosed with approximately 39,000 missed cases yearly, as reported by the EUCLID study, due to unawareness of the condition, unawareness of when to request a CDI test and nonoptimal testing strategies [5, 6]. Therefore, the true CDI burden on the healthcare system remains unknown. However, together with the emergence of hypervirulent *C. difficile* strains (e.g., 027/NAP1 strain), this CDI problematic has fueled the development of (i) new technologies for diagnosing CDI, (ii) new therapeutic strategies, and (iii) optimized protocols for CDI infection prevention and control, to reduce the CDI-associated healthcare burden [7, 8].

To better understand the complex CDI problem, the European Society of Clinical Microbiology and Infectious Diseases (ESCMID) established a workgroup of international experts to define uniform European guidelines. Importantly, these guidelines provide detailed diagnostic guidance for routine detection of *C. difficile* [9]. Recently, the guidelines were updated to incorporate more novel technological developments, specifically to cover the expansion in available nucleic acid amplification techniques (NAAT) [10]. Overall, it was concluded that a two-step algorithm, consisting of a highly sensitive screening test followed by a more specific confirmatory test detecting free toxins, is required because stand-alone methods failed to reach adequate positive predictor values [10]. In addition, the inability to differentiate CDI patients from *C. difficile* carriers when testing for the presence of a toxigenic strain rather than free toxins further limited the use of certain stand-alone techniques. Further, defining optimal diagnostic pathways requires the incorporation of both the performance and the effectiveness of testing (i.e., time to result, workflow, etc.). However, it remains difficult to quantify these aspects, and often there is a lack of sufficient literature data. Nonetheless, toxigenic culture and cell culture cytotoxicity neutralization assay (CCNA) were identified as not suitable for routine application due to their complexity and long turnaround time [11].

In parallel with the emergence of European CDI guidelines, the healthcare system has been steadily restructuring. The upcoming trend (as described by the WHO) of both centralization and decentralization arose from continuous pressure on the healthcare system due to aging of the population and the economic restraints on the health sector. This restructuring has since fueled the emergence of larger centralized laboratories, serving multiple hospitals simultaneously, to improve the efficiency of laboratory testing. However, a centralized lab environment has the disadvantage that it creates a delay in result reporting due to the requirement of specimen transport. This opens the door for the implementation of more advanced technologies. With respect to CDI, the delay to result imposed by transport of the specimen to the laboratory followed by a multistep diagnostic approach in the laboratory might be detrimental to CDI outcome [12]. This does not only impact infection prevention and control measurements but proper patient management in general. Recent reports regarding the increase in CDI incidence hint toward an unresolved issue and the need for further improvement in the efficiency of CDI diagnostics and infection prevention and control measurements [13–15].

In contrast to the centralization of lab infrastructure, a separate and opposite field of point-of-care testing (POCT) has emerged simultaneously. One of the purposes of POCT platforms is to decentralize certain parts of central laboratory testing to reduce time to result and improve clinical decision making. Here, we report the results of a multicenter study in Europe for identification of toxigenic *C. difficile* strains using a new molecular platform called revogene™ from GenePOC Inc. (Quebec, Canada), recently acquired by Meridian Biosciences, that might suit as POCT platform with the right test menu in the future.

## Methods

### Study design

From January to December 2017, the manufacturer coordinated the data collection and analysis of different local microbiology laboratories and national and/or regional reference laboratories for *Clostridioides difficile* across Europe. In total, six laboratories (representatives of the following countries: (i) France, (ii) the Netherlands, (iii) Belgium, (iv) the UK, (v) Switzerland, and (vi) Italy) participated in this study with the purpose of evaluating the revogene™ *C. difficile* assay (abbreviated to “revogene” in the data analysis) in comparison to their routine diagnostic methods. The different laboratories were unaware of the participation of the other laboratories for the duration of their study. The participating laboratories were carefully chosen to obtain sufficient data from various CDI approaches used in Europe for detection of toxigenic *C. difficile* and CDI. The difference in the workflows used as reference was necessary for a well-rounded evaluation of the system, considering the gold standard ESCMID defined for CDI identification is different from the algorithm it recommends for actual CDI diagnosis, as it is too slow [9, 10]. This particular site selection therefore allowed a performance evaluation in terms of accuracy of identification both in the absolute and within a time-sensitive frame.

All evaluation sites collected unformed stool specimens, in a prospective manner, from their routine for the duration of their respective study. Routine testing was mostly performed in parallel to the revogene assay to avoid any bias as consequence of prolonged specimen storage. Collection, transport, and handling of the stool specimens occurred according to the local guidelines while in accordance with the specifications of the different manufacturers. For all the evaluation sites, the manufacturer provided the necessary equipment to work with the revogene *C. difficile* assay.

Of note, in Switzerland, two distinct sites participated in the study design: (i) Bioanalytica (oversaw data management) and (ii) Lucerne Cantonal Hospital (all specimens were transported to Bioanalytica for testing with the revogene, which was performed within 5 days of original specimen collection). Furthermore, Bioanalytica collected stool specimens from two subpopulations of patients: (i) patients suspected of CDI and (ii) outpatients not suspected of CDI. For the latter population, stool specimens from outpatients with a negative BD MAX™ Enteric Bacterial panel (BD) were selected for testing. The epidemiological findings of this investigation fall outside the scope of this publication. However, all specimens were tested with the proper methodology to be included in the performance calculations.

### Definitions

As none of the microbiology laboratories used exactly the same methodology, either due to the fact that they used different CDI diagnostics algorithms or diagnostic tests from different manufacturers, general definitions were put in place to standardize the data analysis. Two main definitions were adopted: (i) CDI cases and (ii) *C. difficile* carriers (or “carriers” in short). A CDI case was defined when free toxin was detected directly from stool specimens. A *C. difficile* carrier was defined by the presence of a toxigenic strain in stools without any detectable free toxin.

For two sites (France and the Netherlands), the gold standards used to define CDI cases and *C. difficile* carriers were, respectively, CCNA and toxigenic culture, in accord with ESCMID guidelines [9, 10]. As for the remaining four sites (the UK, Belgium, Switzerland, and Italy), all used as gold standard different variations of the diagnostic algorithm currently recommended as a suitable, faster alternative to CCNA and toxigenic culture [10]. In general, stool samples found positive for toxin A/B to enzyme immunoassay (EIA) were considered CDI cases. On the other hand, *C. difficile* carriers were defined for EIA-negative specimens in which either the presence of toxin genes could be demonstrated through NAAT testing or toxigenic culture–proved positive.

A detailed overview of the different testing algorithms and commercial tests used at the different sites is provided in Table 1.

**Table 1:** Overview of *C. difficile* testing algorithm

Site	GDH	TOX	NAAT	TC	CCNA	Discrepancies
France	C. DIFF QUIK CHEK (Abbott)		Xpert *C. difficile* (Cepheid)	ChromID plates (bioMerieux)	MRC-5 cells	Enriched toxigenic culture
The Netherlands	N.T.	N.T.	BD MAX CDiff (BD)	ChromID plates (bioMerieux)	MRC-T cells	N.T.
UK	VIDAS® *C. difficile* GDH (bioMerieux)	VIDAS® *C. difficile* TOX A&B (bioMerieux)	Xpert *C. difficile* (Cepheid)	N.T.	N.T.	N.T.
Belgium	Liaison® *C. difficile* GDH (DiaSorin)	Liaison® *C. difficile* TOX A&B (DiaSorin)	Simplexa™ *C. difficile* Universal direct (DiaSorin)	ChromID plates (bioMerieux)	N.T.	N.T.
Switzerland 1	C. DIFF QUIK CHEK (Abbott)		BD MAX CDiff (BD)	N.T.	N.T.	AmpliVue *C. difficile* (Quidel)
Switzerland 2	C. DIFF QUIK CHEK (Abbott)		BD MAX CDiff (BD)	N.T.	N.T.	AmpliVue *C. difficile* (Quidel)
Switzerland 3	C. DIFF QUIK CHEK (Abbott)		Xpert *C. difficile* (Cepheid)	N.T.	N.T.	BD MAX CDiff (BD) and AmpliVue *C. difficile* (Quidel)
Italy	Liaison® *C. difficile* GDH (DiaSorin)	Liaison® *C. difficile* TOX A&B (DiaSorin)	Xpert *C. difficile* (Cepheid)	N.T.	N.T.	TC using ChromID plates (bioMerieux)

*GDH* glutamate dehydrogenase assay; *TOX* rapid assay for toxin A and/or B detection; *NAAT* nucleic acid amplification test; *TC* toxigenic culture; *CCNA* cell cytotoxicity neutralization assay; *N.T.* not tested

### Discrepancy analysis

In case of discrepant results between the revogene and the routine methodology (and/or golden standard method), additional testing was performed. Table 1 provides an overview of the methodology used to resolve the discrepancies at each site (when available).

### Data analysis and statistics

Sensitivity, specificities, and positive and negative predictive values are expressed as percentages. Confidence intervals were determined with Clopper-Pearson method.

## Results

### Inclusion rate and valid results

A total of 2662 unformed stool specimens were prospectively collected and tested with their routine CDI diagnostic algorithm, in parallel with the revogene, across six different evaluation sites in Europe. A total of 41 specimens (1.5%) were excluded from further analysis of which 21 (0.79%) due to invalid revogene results, 2 (0.08%) due to testing of external controls, 7 (0.26%) due to not in agreement with the manufacturer’s specifications, and 11 (0.41%) due to lack of sufficient reference data. For the 2621 specimens that were included in the analysis, 4.2% displayed an unresolved/indeterminate result with the revogene assay on initial testing, but after repetition, all specimens gave a valid result.

### Site by site results

Table 2 provides a detailed overview of the performance characteristics for each site that participated in the multicenter study for identification of (i) CDI and (ii) *tcdB*+ strains, which is the sum of the number of CDI cases and the number of *C. difficile* carriers. Sensitivity/specificity of the revogene *C. difficile* assay for identification of CDI fluctuated between 85–100% and 97–100%, respectively. For the identification of *tcdB*+ strains, sensitivity/specificity fluctuated between 84–100% and 96–100%, respectively.

**Table 2:** Study site overview and performance

**CDI**											
Site	# Specimens	# CDI	Study period	Sensitivity [95% CI]				Specificity [95% CI]			
FR	308	19	01–03 2017	100%	[82.4	–	100]	99.7%	[98.1	–	100]
NL	386	25	01–04/2017	96.0%	[79.7	–	99.9]	98.9%	[97.2	–	99.7]
UK	296	15	01–06/2017	100%	[78.2	–	100]	98.2%	[95.9	–	99.4]
BE	189	22	08–11/2017	90.9%	[70.8	–	98.9]	97.6%	[94.0	–	99.3]
CH1	232	22	03–09/2017	100%	[84.6	–	100]	100%	[98.3	–	100]
CH2	504	5	06–09/2017	100%	[47.8	–	100]	99.6%	[98.6	–	99.6]
CH3	298	14	03–06/2017	85.7%	[57.2	–	98.2]	98.6%	[96.4	–	99.6]
IT	408	48	09–11/2017	100%	[92.6	–	100]	98.3%	[96.4	–	99.4]
***tcdB*+ strains**											
Site	# Specimens	# *tcdB*+	Study period	Sensitivity [95% CI]				Specificity [95% CI]			
FR	308	35	01–03/2017	91.4%	[76.9	–	98.2]	99.6%	[98.0	–	99.9]
NL	386	28	01–04/2017	89.3%	[71.8	–	97.7]	98.9%	[97.2	–	99.7]
UK	296	43	01–06/2017	95.3%	[84.2	–	99.4]	98.0%	[95.5	–	99.4]
BE	189	36	08–11/2017	86.1%	[70.5	–	95.3]	96.1%	[91.7	–	98.6]
CH1	232	38	03–09/2017	100%	[90.8	–	100]	100%	[98.1	–	100]
CH2	504	11	06–09/2017	100%	[47.8	–	100]	99.6%	[98.5	–	99.5]
CH3	298	19	03–06/2017	84.2%	[60.4	–	96.6]	98.6%	[96.4	–	99.6]
IT	408	89	09–11/2017	91.0%	[83.1	–	96.0]	98.1%	[96.0	–	99.3]

*FR* France; *NL* the Netherlands; *UK* the United Kingdom; *BE* Belgium; *CH* Switzerland; *IT* Italy. The numbering 1 to 3 for Switzerland reflects the different evaluations setup as described in the Method section (i.e., Bioanalytica, patients suspected of CDI; Bioanalytica, outpatients not suspected of CDI; and Lucerne Cantonal hospital, respectively). Sensitivities/specificities are reported with their respective 95% confidence interval (CI)

*CDI Clostridioides difficile* infection; *tcdB*+, a *Clostridioides difficile* identified to carry the *tcdB* gene

### Revogene *C. difficile* overall performance

#### Identification of CDI and *tcdB*+ strains

**Fig. 1:**
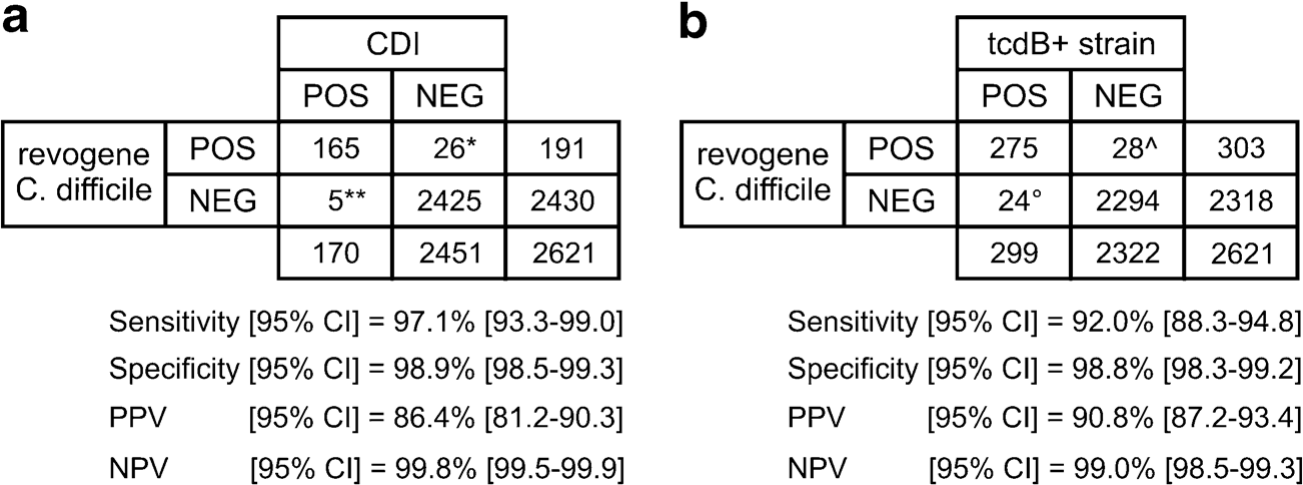
Overall performance characteristics of the revogene assay for identification of CDI and *tcdB*+ strains. **a** Performance characteristics of revogene for identifying CDI cases. *15/26 false positives were found to also be positive with additional techniques as described in the method section. **3/5 false negatives were also found to be negative using the same discrepancy analysis. **b** Performance characteristics of revogene for identifying *tcdB*+ strains. ^16/28 false positives were found to be a true positive after discrepancy analysis as described in the method section. °11/24 false negatives were confirmed as true negative using the same discrepancy analysis

According to the definitions described in the Methods section, Fig. 1 provides an overview of pooled performance of the revogene assay (as stand-alone method) for identification of CDI cases and *tcdB*+ strains. Overall, the revogene assay performed well with a sensitivity/specificity of 97.1% [93.3–99.0] and 98.9% [98.4–99.2] for identifying CDI cases and 91.9% [88.2–94.8] and 98.7% [98.2–99.1] for identifying *tcdB*+ strains. After resolving discrepancies with additional testing, the sensitivity/specificity improved to 98.8% [95.8–99.9] and 99.6% [99.2–99.8] for identifying CDI cases and to 95.7% [92.8–97.7] and 99.4% [99.0–99.8] for identification of *tcdB*+ stains.

#### Comparison to toxigenic culture

**Fig. 2:**
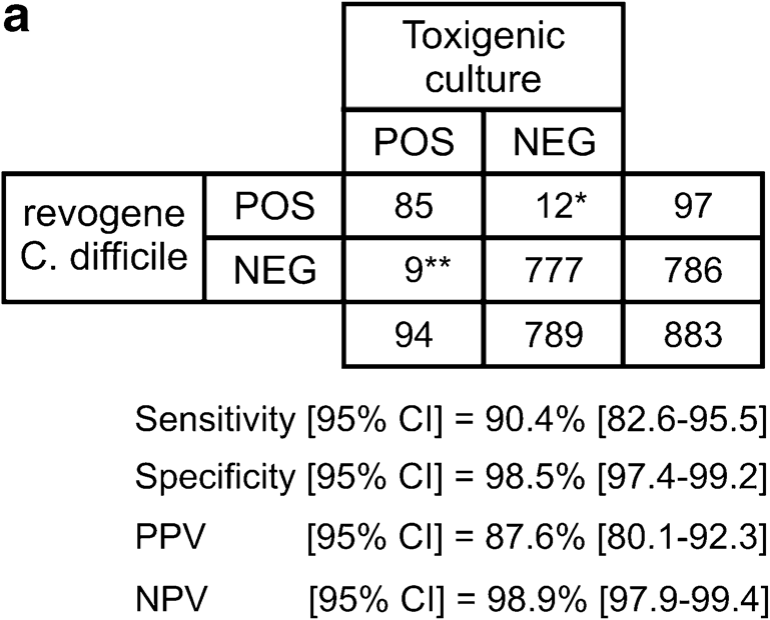
Overall performance characteristics of the revogene assay in comparison to toxigenic culture. **a** Performance characteristics of revogene with: *8/12 false positives were found to be a true positive after discrepancy analysis as described in the method section. **2/9 false negatives were confirmed as true negative using the same discrepancy analysis

Three of the six evaluation sites performed toxigenic culture on all specimens tested which allowed us to compare the revogene assay against this gold standard method (Fig. 2). Overall, the revogene assay performed well with sensitivity/specificity of 90.4% [82.6–95.5] and 98.5% [97.4–99.2], respectively. This performance corresponded to a positive and negative predictive value (PPV/NPV) of 87.6% [80.1–92.3] and 98.9% [97.9–99.4], respectively, with a reported overall prevalence of 10.6% [8.7–12.9]. After resolving the discrepancies, the sensitivity, specificity, PPV, and NPV improved to 93.0 [86.1–97.1], 99.5 [98.7–99.9], 95.9 [89.7–98.4], and 99.1% [98.2–99.6], respectively.

#### Revogene assay as part of a multistep algorithm

In the above analysis, the revogene performance is determined as stand-alone method against the reference method. However, the European Society of Clinical Microbiology and Infectious Diseases (ESCMID) does not recommend the use of a single commercial test as stand-alone test for diagnosing CDI [9]. Therefore, we extended our data analysis to incorporate the revogene assay as part of a multistep (i.e., two- or optional three-steps) algorithm, with the revogene assay either at the (i) front of the algorithm, to be tested on all specimens (i.e., revogene -TOX), or (ii) at the end of the algorithm, as confirmatory test (GDH-TOX-revogene).

**Fig. 3:**
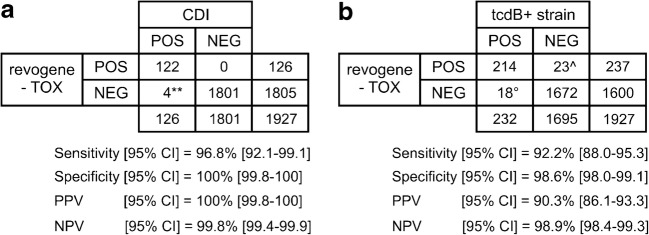
Overall performance characteristics of the revogene-TOX algorithm. **a** Performance characteristics of revogene-TOX algorithm for identifying CDI cases. **1/4 false negatives were confirmed as true negative using the discrepancy analysis. **b** Performance characteristics of revogene-TOX algorithm for identifying *tcdB*+ strains. ^12/23 false positives were found to be a true positive after discrepancy analysis as described in the method section. °8/18 false negatives were confirmed as true negative using the same discrepancy analysis

**Fig. 4:**
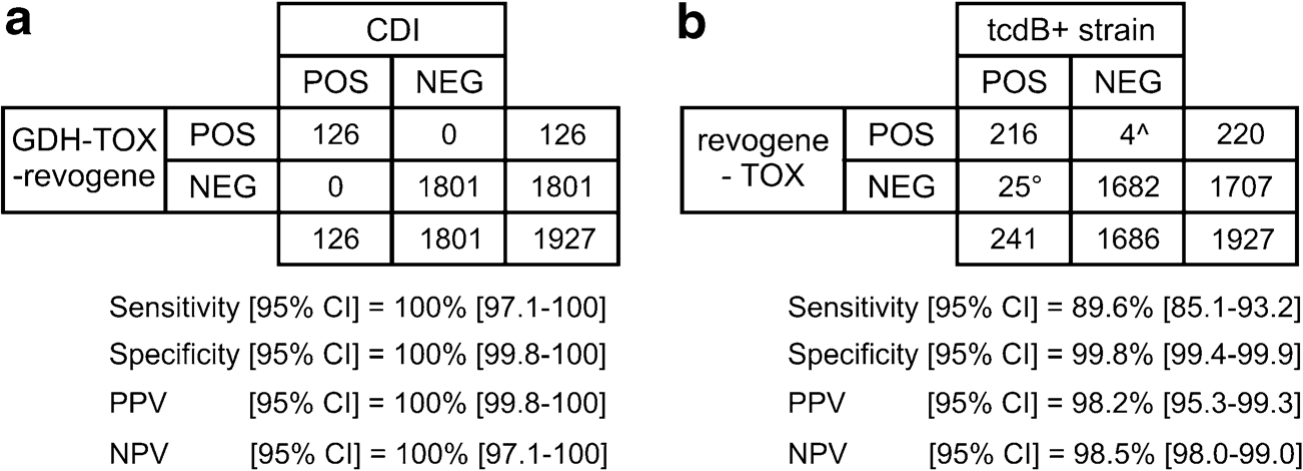
Overall performance characteristics of the GDH-TOX-revogene algorithm. **a** Performance characteristics of GDH-TOX-revogene algorithm for identifying CDI cases. **b** Performance characteristics of GDH-TOX-revogene algorithm for identifying *tcdB*+ strains. ^3/4 false positives were found to be a true positive after discrepancy analysis as described in the method section. °6/25 false negatives were confirmed as true negative using the same discrepancy analysis

Overall, the revogene-TOX algorithm performed well with a sensitivity/specificity of 96.8% [92.1–99.1] and 100% [99.8–100] for identifying CDI cases and 92.2% [88.0–95.3] and 98.6% [98.0–99.1] for identifying *tcdB*+ strains (Fig. 3). Resolution of the discrepancies improved the sensitivity/specificity performance to 97.6% [93.2–99.5] and 100% for identifying CDI cases and 95.8% [92.4–98.0] and 99.3% [98.8–99.7] for identifying *tcdB*+ strains. On the other hand, the GDH-TOX-revogene algorithm performed similarly with a sensitivity/specificity of 100% [97.1–100] and 100% [99.8–100] for identifying CDI cases and 89.6% [85.1–93.2] and 99.8% [99.4–99.9] for identifying *tcdB*+ strains (Fig. 4). Resolution of the discrepancies improved the sensitivity/specificity performance to 92.0% [87.8–95.1] and 99.9% [99.7–100] for identifying *tcdB*+ strains.

## Discussion

Despite significant effort and various strategies to improve CDI diagnostics, *C. difficile* remains a global healthcare burden that keeps challenging all stakeholders, from nurse to clinical microbiologist, to improve in their current field to overcome this global threat. At the beginning of the century, molecular biology techniques opened the door to improved diagnostics, but despite the enormous progression made in that field, molecular diagnostic techniques (like NAAT) remain underutilized when it comes to diagnosing CDI. Recently, the amount of available molecular NAAT assays for detection of CDI have vastly expanded which made the technology more accessible, especially in the central laboratory, and competitive in terms of pricing. However, with the emergence of POCT testing, NAAT technology for infectious diseases is now also more accessible to smaller labs, or it could be used to equip satellite labs connected to a central lab for urgent testing. In this work, we report the results from a multicenter *Clostridioides difficile* study that assessed the performance of a novel molecular platform (i.e., revogene™) from Meridian Biosciences Canada Inc. (Quebec, Canada). The revogene assay targets the *tcdB* gene of toxigenic *C. difficile* and required minimal hands-on time, and results were available in approximately an hour. The 1 to 8 flexible throughput combined with the ease of use would allow this platform to be implemented in both small satellite and central labs.

In this multicenter study, the revogene assay was capable of adequately identifying CDI cases and *tcdB*+ strains. However, current diagnostic guidelines in Europe recommend to perform a two-step algorithm for the identification of CDI cases [10]. In contrast, the recently updated clinical practice guidelines for CDI (by the IDSA and SHEA societies) state that it would be acceptable to use a stand-alone NAAT assay for diagnosis CDI under the condition that pre-defined criteria for stool specimen submission are used by clinicians and laboratory personnel [16]. Interestingly, here, we only observed minor differences between the revogene assay (stand-alone) and revogene-TOX algorithm in terms of correctly identifying CDI cases and *tcdB*+ strains. Due to the fact that in both scenarios (i.e., revogene and revogene-TOX) the same dataset was analyzed with the same definition for CDI cases and *tcdB*+ carriers, this might raise doubt on the usefulness of the confirmatory TOX test in such a two-step algorithm. However, it must be noted that the absence of the TOX test would not allow differentiation of CDI from asymptomatic carriage of *C. difficile*. When comparing the revogene-TOX and GDH-TOX-revogene algorithm, we also only observed some minor differences in performance which raises the general question about how to best define the balance between algorithm performance and time to result. However, this topic falls out of the scope of the current work, and additional research is required to answer this question.

A major strength of this work was the multicenter approach that allowed us to (i) capture data on the different *C. difficile* diagnostic methodologies used across Europe and (ii) gather an extensive dataset to determine the performance characteristics of the revogene assay. The work was further strengthened due to standardization of the dataset through the use of definitions for CDI and *tcdB*+ strains. This allowed us to compare different diagnostic algorithms for CDI under the same conditions. However, the fact that different CDI diagnostic methodologies were used and standardization of the data was required also imposes a limitation to the study as the results cannot be extrapolated and properly compared to the current available literature.

An added value of this system is a decrease in the turnaround time (TAT): This was extremely significant in the Belgian and French sites (23–47-h reduction), which are reference centers where culturing is conducted for epidemiological reasons; nonetheless, sites which already used workflows considered time-accurate for CDI diagnosis showed a decrease in TAT, if less marked (1 h for the Netherlands and Switzerland, 2 h for Italy and UK).

In conclusion, the revogene *C. difficile* molecular assay for detection of the *tcdB* gene of toxigenic *Clostridioides difficile* is a high quality and simple to use diagnostic test providing reliable results in approximately an hour. This makes the revogene assay suitable for implementation as diagnostic utility, either as frontline/confirmatory test in a multistep algorithm or as stand-alone method if used with proper institutional criteria for collection of CDI-suspected stool specimens.

## References

[1] Zarb P, Coignard B, Griskeviciene J, Muller A, Vankerckhoven V, Weist K et al (2012) The European Centre for Disease Prevention and Control (ECDC) pilot point prevalence survey of healthcare-associated infections and antimicrobial use. Euro Surveill 17(46) 10.2807/ese.17.46.20316-en 23171822

[2] Kuijper EJ, Coignard B, Tüll P, ESCMID Study group for Clostridium difficile; EU Member States; European Centre for Disease Prevention and Control (2006). Emergence of *Clostridium difficile*-associated disease in North America and Europe. Clin Microbiol Infect.

[3] Bouza E (2012). Consequences of Clostridium difficile infection: understanding the healthcare burden. Clin Microbiol Infect.

[4] HOPE (2013) *Clostridium difficile* infection in Europe, a CDI Europe report. Available from: http://www.multivu.com/assets/60637/documents/60637-CDI-HCP-Report-original.pdf

[5] Davies KA, Longshaw CM, Davis GL, Bouza E, Barbut F, Barna Z (2014). Underdiagnosis of Clostridium difficile across Europe: the European, multicentre, prospective, biannual, point-prevalence study of Clostridium difficile infection in hospitalized patients with diarrhea (EUCLID). Lancet Infect Dis.

[6] Acalá L, Martín A, Martín M, Sánchez-Somolinos M, Catalân P, Pelâez T (2012). The undiagnosed cases of Clostridium difficile infection in a whole nation: where is the problem?. Clin Microbiol Infect.

[7] Ooijevaar RE, van Beurden YH, Terveer EM, Goorhuis A, Bauer MP, Keller JJ et al (2018) Update of treatment algorithms for Clostridium difficile infection. Clin Mircobiol Infect. 10.1016/j.cmi.2017.12.022 10.1016/j.cmi.2017.12.022 29309934

[8] Krutova M, Kinross P, Barbut F, Hajdu A, Wilcox M, Kuijper E (2017) How to: surveillance of Clostridium difficile infections. Clin Microbiol Infect. 10.1016/j.cmi.2017.12.008 10.1016/j.cmi.2017.12.008 29274463

[9] Crobach MJ, Dekkers OM, Wilcox MH, Kuijper EJ (2009). European Society of Clinical Microbiology and Infectious Diseases (ESCMID): data review and recommendations for diagnosing Clostridium difficile-infection (CDI). Clin Microbiol Infect.

[10] Crobach MJ, Planche T, Eckert C, Barbut F, Terveer EM, Dekkers OM (2016). European Society of Clinical Microbiology and Infectious Diseases: update of the diagnostic guidance document for *Clostridium difficile* infection. Clin Microbiol Infect.

[11] Van Dorp SM, Notermans DW, Alblas J, Gastmeier P, Mentula S, Nagy E et al (2016) Survey of diagnostic and typing capacity for *Clostridium difficile* infection in Europe, 2011 and 2014. Euro Surveill 21(29). 10.2807/1560-7917.ES.2016.21.29.30292 10.2807/1560-7917.ES.2016.21.29.30292 27469624

[12] Barbut F, Surgers L, Eckert C, Visseaux B, Cuignet M, Mesquita C (2014). Does a rapid diagnosis of Clostridium difficile infection impact on quality of patient management?. Clin Microbiol Infect.

[13] Alicino C, Giacobbe DR, Durando P, Bellina D, DI Bella AM, Paganino C (2016). Increasing incidence of Clostridium difficile infections: result from a 5-year retrospective study in a large teaching hospital in the Italian region with the oldest population. Epidemiol Infect.

[14] Vindigni SM, Surawicz CM (2015). C. difficile infection: changing epidemiology and management paradigms. Clin Transl Gastroenterol.

[15] Barbut F, Ramé L, Petit A, Suzon L, de Chevigny A, Eckert C (2015). Prevalence of Clostridium difficile infection in hospitalized patients with diarrhea: results of a French prospective multicenter bi-annual point prevalence study. Presse Med.

[16] McDonald LC, Gerding DN, Johnson S, Bakken JS, Carroll KC, Dubberke ER (2018). Clinical practice guidelines for Clostridium difficile infection in adults and children: 2017 update by the Infectious Diseases Society of America (IDSA) and Society for Healthcare Epidemiology of America (SHEA). Clin Infect Dis.

